# Postoperative Antibiotic Duration and Treatment Failure After Aseptic Revision Arthroplasty With a Single Unexpected Positive Culture: A Retrospective Cohort Study

**DOI:** 10.1111/os.70452

**Published:** 2026-10-21

**Authors:** Hao‐Chang Hsieh, Feng‐Chih Kuo, Allen Herng Shouh Hsu, Shih‐Hsiang Yen, Po‐Chun Lin, Cheng‐Ta Wu

**Affiliations:** ^1^ Department of Orthopedic Surgery E‐Da Hospital/I‐Shou University Kaohsiung, Taiwan China; ^2^ Department of Orthopaedic Surgery Kaohsiung Chang Gung Memorial Hospital Kaohsiung, Taiwan China; ^3^ College of Medicine Chang Gung University Kaohsiung, Taiwan China; ^4^ Center for General Education Cheng Shiu University Kaohsiung, Taiwan China

**Keywords:** antibiotic duration, aseptic revision, unexpected positive intraoperative culture

## Abstract

**Objectives:**

The clinical significance of a single unexpected positive intraoperative culture (UPC) during aseptic revision arthroplasty remains unclear. This study primarily aimed to evaluate the association between postoperative antibiotic duration and treatment failure in patients with a single UPC after aseptic revision total hip or knee arthroplasty. A secondary aim was to compare failure‐free survival between patients with a single UPC and those with multiple UPCs.

**Methods:**

We retrospectively reviewed 51 presumed aseptic revision hip or knee arthroplasties with UPCs performed between January 1999 and December 2023. Cases meeting the 2018 ICM criteria for periprosthetic joint infection were excluded. Postoperative antibiotic duration was the exposure of interest. Treatment failure, defined as infection‐related re‐revision, was the primary outcome; failure‐free survival was the secondary outcome. Continuous variables were compared using the Mann–Whitney *U* test, and categorical variables were compared using chi‐square or Fisher's exact tests. Factors associated with failure were examined using univariate logistic regression, and failure‐free survival was assessed using Kaplan–Meier analysis with the log‐rank test.

**Results:**

Among 39 patients with one UPC, treatment failure occurred in 18%. Antibiotic courses longer than 21 days were associated with failure (OR 28.1). In the small subgroup with available data (*n* = 13), synovial biomarkers did not show a statistically significant difference between success and failure groups. Patients with one UPC had better failure‐free survival than those with ≥ 2 UPCs.

**Conclusion:**

The observed association between prolonged antibiotic therapy and treatment failure may reflect underlying infection severity rather than a causal effect of antibiotic duration. Individualized management is recommended for UPCs in aseptic revision arthroplasty.

## Graphical abstract

Prolonged postoperative antibiotic use was associated with higher treatment failure in aseptic revision arthroplasty with a single unexpected positive culture, suggesting that extended antibiotic therapy may not improve outcomes and highlighting the need for individualized treatment strategies.

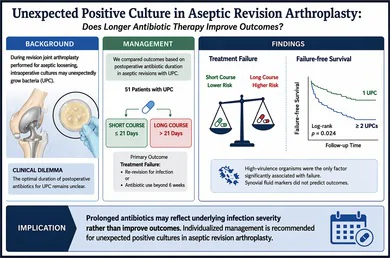

**Abbreviations**
CCI: Charlson Comorbidity Index
ICM: International Consensus Meeting
IQR: interquartile range
PJI: periprosthetic joint infection
PMN: polymorphonuclear neutrophil
THA: total hip arthroplasty
TJA: total joint arthroplasty
TKA: total knee arthroplasty
UPC: unexpected positive intraoperative culture
WBC: white blood cell

## 1 Introduction

Revision total joint arthroplasty (TJA) is increasing worldwide. In the United States, revision total hip arthroplasty (THA) and revision total knee arthroplasty (TKA) are projected to increase by 43%–70% and 78%–182%, respectively, from 2014 to 2030 [1]. Aseptic loosening remains the main indication for revision [2], yet differentiating it from low‐grade periprosthetic joint infection (PJI) continues to pose a diagnostic challenge [3].

Despite advances in preoperative evaluation, intraoperative cultures may still yield unexpected positive cultures (UPCs) in 6%–12% of revisions initially classified as aseptic [4, 5]. A recent systematic review and meta‐analysis of 7449 presumed aseptic revision THAs reported a UPC prevalence of 15.1% and found higher risks of all‐cause re‐revision and subsequent PJI in patients with UPCs [6]. According to the 2018 International Consensus Meeting (ICM) criteria, two positive cultures of the same organism constitute a major criterion for PJI [7]. However, the relevance of a single positive culture (1 UPC) remains controversial.

Traditionally regarded as contamination, a single unexpected positive intraoperative culture (1 UPC) has resulted in wide variation in postoperative management, ranging from observation to prolonged antibiotic therapy. However, growing evidence indicates that 1 UPC is not always benign. Several studies have shown that even in presumed aseptic revisions, 1 UPC may be associated with reinfection or re‐revision, particularly when high‐virulence organisms are present [8, 9, 10]. These data challenge the assumption of contamination and highlight the need for individualized management. Consequently, treatment strategies remain inconsistent, with some surgeons prescribing extended antibiotics and others adopting a conservative approach for low‐virulence organisms. The optimal duration of antibiotic therapy in this setting remains undefined.

Because multiple positive cultures are more suggestive of PJI, a single UPC represents the most diagnostically ambiguous subgroup and has variable postoperative management. Therefore, the primary objective of this study was to evaluate whether postoperative antibiotic duration was associated with treatment failure in patients with a single UPC after aseptic revision THA or TKA. The secondary objective was to compare failure‐free survival between patients with a single UPC and those with multiple UPCs. We hypothesized that prolonged antibiotic therapy may influence failure risk and that a single UPC does not necessarily represent contamination.

## 2 Methods

This study was approved by the ethics committee of the Chang Gung Medical Foundation, with the approval reference number (IRB No.: 202101507B0C601, date of approval: 2023/3/8). Informed consent was waived by the ethics committee of Chang Gung Medical Foundation. After Institutional Review Board approval, this retrospective study was conducted at a tertiary referral center. From January 1999 to December 2023, 2932 revision total hip and knee arthroplasty procedures were performed. Patients were eligible if they underwent revision for presumed aseptic indications and had at least one UPC. Exclusion criteria included PJI based on the 2018 ICM criteria, megaprosthesis implantation, negative cultures, incomplete data, or follow‐up < 1 year in patients without treatment failure. Cases were identified through surgical records and microbiology databases. After applying these criteria, 747 PJI cases, 94 megaprosthesis cases, 2010 culture‐negative revisions, 7 patients lost to follow‐up before 1 year, and 23 with missing data were excluded. The final cohort included 51 UPC‐positive revisions initially classified as aseptic (Figure 1). Clinical variables collected included age, sex, body mass index, joint involved, laterality, Charlson Comorbidity Index (CCI), comorbidities, American Society of Anesthesiologists (ASA) classification, indication for revision, serum inflammatory markers, and synovial fluid parameters when available.

**FIGURE 1:**
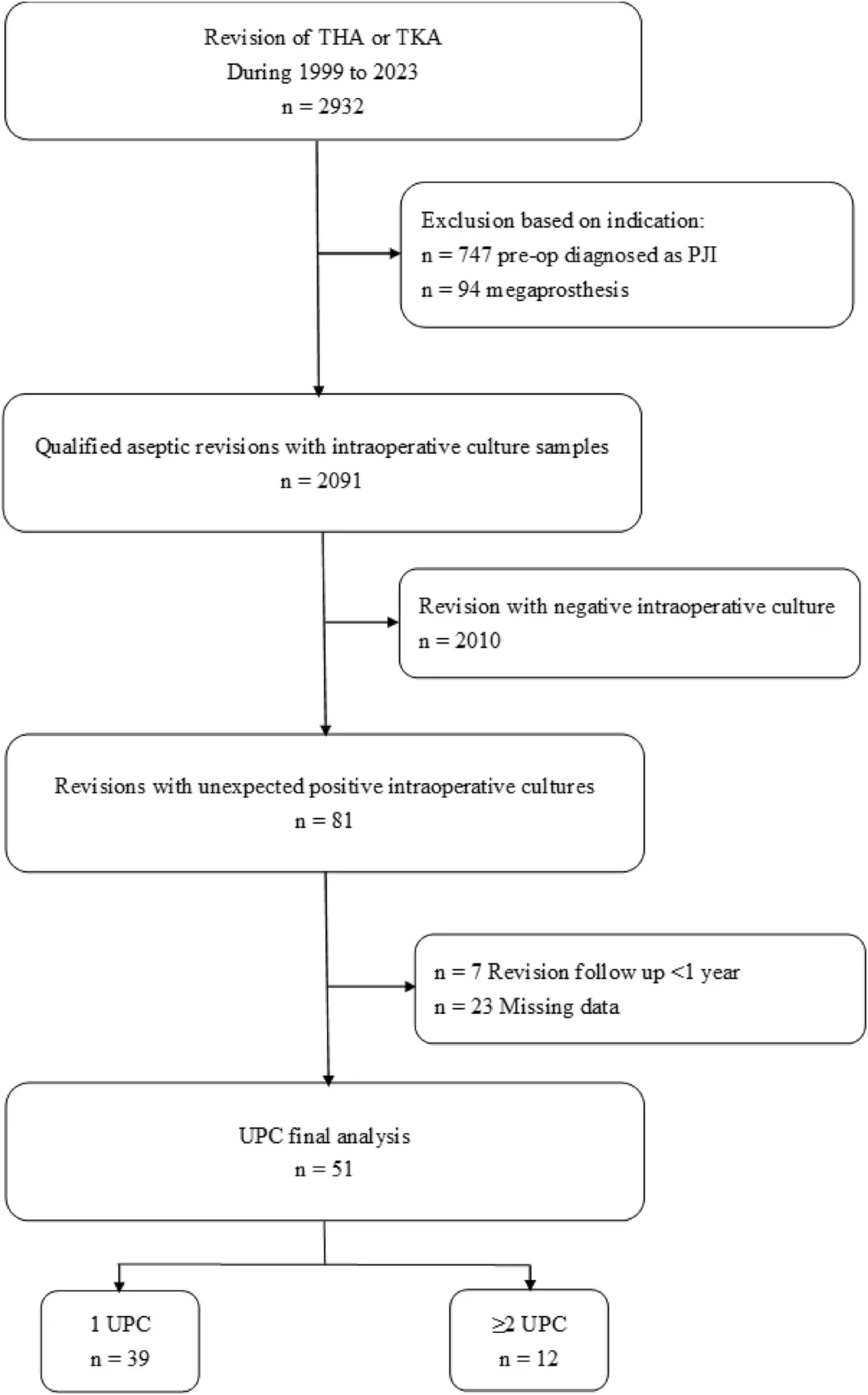
Flow diagram of patient selection and inclusion in the final study cohort.

Aspiration was performed preoperatively or intraoperatively when elevated inflammatory markers or clinical suspicion of infection was present, following 2018 ICM recommendations. Synovial fluid analysis was available in 13 of the 39 patients in the single‐UPC subgroup. Because arthrocentesis was performed selectively rather than routinely, these patients represented a nonrandom subset. Synovial fluid was analyzed for white blood cell (WBC) count and polymorphonuclear neutrophil (PMN) percentage, with diagnostic thresholds of WBC > 3000/μL and PMN > 80%.

During revision surgery, three to five periprosthetic tissue samples were collected for microbiological culture. All specimens underwent aerobic and anaerobic incubation for up to 14 days. A single UPC was defined as growth of a microorganism from only one intraoperative tissue sample obtained during the index revision procedure, whereas multiple UPCs were defined as growth from two or more intraoperative tissue samples obtained during the same index revision procedure. Revisions initially classified as aseptic that yielded any positive intraoperative culture were designated as UPC cases. Following the identification of a UPC, postoperative antibiotic management was non‐standardized and left to surgeon discretion based on organism virulence, serial inflammatory markers, wound healing, and clinical suspicion. Antibiotic agents, dosage, and route of administration were available from the medical records but were highly heterogeneous. Given the small sample size and sparse regimen‐specific subgroups, these variables were not formally analyzed, and the study focused on postoperative antibiotic duration.

### 2.1 Statistical Analysis

The primary outcome was treatment failure, defined strictly as re‐revision for infection. The secondary outcome was failure‐free survival, measured from revision surgery to treatment failure or last follow‐up.

All statistical analyses were performed using SPSS version 26.0 (IBM Corp., Armonk, NY, USA). Because continuous variables were non‐normally distributed on Shapiro–Wilk testing (*p* < 0.05), they are reported as median and interquartile range (IQR). Continuous variables were compared using the Mann–Whitney *U* test. Categorical variables were compared using chi‐square or Fisher's exact tests. Univariate logistic regression analysis was used to identify factors associated with treatment failure. Failure‐free survival between the 1 UPC and ≥ 2 UPC groups was compared using the Kaplan–Meier method with the log‐rank test. Patients who did not experience infection‐related re‐revision were censored at their last available follow‐up or at 60 months, whichever occurred first. Statistical significance was set at *p* < 0.05.

## 3 Results

### 3.1 Baseline Characteristics and Clinical Findings

Baseline demographic and surgical characteristics of the 51 patients are summarized in Table 1. The median age was 61 years (IQR, 55–71), and 57% were male. Most revisions involved the hip (90%), with knees accounting for 10%. Aseptic loosening was the most common indication (51%), followed by osteolysis or polyethylene wear (27.5%) and periprosthetic fracture (17.6%). The median CCI was 3 (IQR 2–4). Among patients with available synovial measurements, 7 of 17 (41.2%) had a WBC count > 3000/μL, and 8 of 16 (50.0%) had a PMN percentage > 80%. Subgroup analysis was performed for the 39 patients with a 1 UPC. Of these, 32 (82%) achieved treatment success, whereas 7 (18%) experienced treatment failure due to infection‐related re‐revision (Table 2).

**TABLE 1:** Baseline demographics and operative characteristics of 51 revision arthroplasties with unexpected positive intraoperative cultures (UPCs).

Variables	
Age, years, median (IQR)	61 (55–71)
Sex, *n* (%)	
Male	29 (57)
Female	22 (43)
BMI (kg/m^2^), median (IQR)	27.3 (23.5–28.6)
Joint, *n* (%)	
Knee	5 (10)
Hip	46 (90)
Laterality, *n* (%)	
Left	25 (49)
Right	26 (51)
CCI score, median (IQR)	3 (2–4)
DM, *n* (%)	10 (19.6)
Renal failure, *n* (%)	5 (9.8)
Liver disease, *n* (%)	9 (17.6)
Total Elixhauser Groups per record, median (IQR)	2 (1–3)
Length of stay, median (IQR)	8 (6–16)
ASA, *n* (%)	
1	0 (0)
2	29 (57)
3	22 (43)
4	0 (0)
Reason for revision, *n* (%)	
Osteolysis + PE wear	14 (27.5)
Aseptic loosening	26 (51.0)
Periprosthetic fracture	9 (17.6)
Dislocation	2 (3.9)
UPC vs. ≥ 2 UPCs, *n* (%)	
1UPC	39 (76.5)
≥ 2UPCs	12 (23.5)
Serum biomarkers	
ESR, > 30 mm/h, *n* (%)	16 (31.4)
CRP, > 10 mg/L, *n* (%)	10 (19.6)
d‐dimer, > 850 mg/L, *n* (%)	20 (39.2)
Cr. (mg/dL), median (IQR)	0.89 (0.72–1.10)
Joint aspirate, *n* (%)	
Synovial fluid WBC > 3000/µL, *n*/*N* (%)	7/17 (41.2)
Synovial PMN > 80%, *n*/*N* (%)	8/16 (50.0)

*Note:* Continuous variables are presented as medians with interquartile ranges (IQR). Categorical variables are presented as number (%). For synovial fluid variables, percentages were calculated using the number of patients with available measurements as the denominator.

Abbreviations: ASA = American Society of Anesthesiologists, BMI = body mass index, CCI = Charlson Comorbidity Index, Cr. = serum creatinine, CRP = C‐reactive protein, DM = diabetes mellitus, ESR = erythrocyte sedimentation rate, PE = polyethylene, PMN = polymorphonuclear neutrophil, Preop = preoperative, UPC = unexpected positive intraoperative culture, WBC = white blood cell.

**TABLE 2:** Clinical outcomes and associated factors in patients with a single UPC.

Variable	Success (*n* = 32)	Failure (*n* = 7)	*p*
Age, years, median (IQR)	60 (56.5–70.75)	64 (55.5–66)	1.000
Sex, *n* (%)			0.678
Male	14 (44)	2 (29)	
Female	18 (56)	5 (71)	
BMI (kg/m^2^), median (IQR)	27.3 (24.3–28.5)	26.5 (23.6–29.7)	0.634
Joint, *n* (%)			1.000
Hip	28 (88)	7 (100)	
Knee	4 (12)	0 (0)	
Laterality, *n* (%)			0.098
Left	17 (53)	1 (14)	
Right	15 (47)	6 (86)	
CCI score, median (IQR)	3 (1–4)	3 (0–6)	1.000
DM, *n* (%)	5 (15)	2 (28)	0.588
Renal failure, *n* (%)	4 (12)	1 (14)	1.000
Liver disease, *n* (%)	7 (22)	0 (0)	0.313
Total elixhauser groups per record, median (IQR)	1 (1–2)	2 (0–4)	0.485
Length of stay, median (IQR)	7 (5–12.2)	9 (7–14.5)	0.270
ASA, *n* (%)			0.415
1	0 (0)	0 (0)	
2	20 (63)	3 (43)	
3	12 (37)	4 (57)	
4	0 (0)	0 (0)	
Reason for revision, *n* (%)			0.182
Osteolysis + PE wear	9 (28)	2 (29)	
Aseptic loosening	16 (50)	4 (57)	
Periprosthetic fracture	7 (22)	0 (0)	
Dislocation	0 (0)	1 (14)	
Serum biomarkers			
ESR, > 30 mm/h, *n* (%)	5 (15)	3 (42)	0.137
CRP, > 10 mg/L, *n* (%)	2 (6)	3 (42)	0.032
d‐dimer, > 850 mg/L, *n* (%)	13 (40)	4 (57)	0.677
Cr. (mg/dL), median (IQR)	0.88 (0.73–1.05)	0.82 (0.66–0.99)	0.631
Synovial fluid WBC > 3000/µL, *n/N* (%)	2/11 (18.2)	1/2 (50.0)	0.423
Synovial PMN > 80%, *n*/*N* (%)	5/11 (45.5)	1/2 (50.0)	1.000

*Note:* Continuous variables are presented as medians with interquartile ranges (IQR). Categorical variables are presented as number (%).

Abbreviations: ASA = American Society of Anesthesiologists, BMI = body mass index, CCI = Charlson Comorbidity Index, Cr. = serum creatinine, CRP = C‐reactive protein, DM = diabetes mellitus, ESR = erythrocyte sedimentation rate, PE = polyethylene, PMN = polymorphonuclear neutrophil, Preop = preoperative, UPC = unexpected positive intraoperative culture, WBC = white blood cell.

Demographic and surgical factors were comparable between the success and failure groups, including age, sex, BMI, and comorbidity variables. Right‐sided procedures were more common in patients with treatment failure (86% vs. 47%, *p* = 0.098). Elevated preoperative CRP (> 10 mg/L) was also more frequent in the failure group (42% vs. 6%, *p* = 0.032). Synovial fluid analysis was available for a limited subset of 13 patients in the single‐UPC subgroup. Within this small subgroup, no statistically significant differences were observed in joint fluid markers between the success and failure groups, though these analyses are highly underpowered. Specifically, elevated WBC (> 3000/μL) occurred in 1 of 2 failures (50.0%) and 2 of 11 successes (18.2%; *p* = 0.423), while elevated PMN (> 80%) was observed in 1 of 2 failures (50.0%) and 5 of 11 successes (45.5%; *p* = 1.000).

The most frequently isolated organisms were *Staphylococcus epidermidis* (8/39, 20.5%), coagulase‐negative *staphylococci* (7/39, 17.9%), and *Cutibacterium acnes* (5/39, 12.8%) (Table 3).

**TABLE 3:** Microorganisms identified in 39 revision arthroplasties with a single UPC.

Variables	Success (*n* = 32)	Failure (*n* = 7)
Microorganism, *n* (%)		
Gram‐positive bacteria		
*S. aureus*	1 (3.1)	0 (0)
*S. epidermidis*	7 (21.9)	1 (14.3)
Coag(−) *staphylococcus*	4 (12.5)	3 (42.9)
*S. mitis*	1 (3.1)	0 (0)
*Enterococcus avium*	1 (3.1)	0 (0)
OSSA	2 (6.3)	1 (14.3)
*Bacillus cereus*	2 (6.3)	1 (14.3)
MRSA	1 (3.1)	0 (0)
*Cutibacterium acnes*	5 (15.6)	0 (0)
*Bifidobacterium adolescentis*	1 (3.1)	0 (0)
Gram‐negative bacteria		
*E. coli*	4 (12.5)	0 (0)
*Moraxella* sp.	0 (0)	1 (14.3)
*P. aeruginosa*	1 (3.1)	0 (0)
*Proteus mirabilis*	1 (3.1)	0 (0)
*K. pneumoniae*	1 (3.1)	0 (0)

*Note:* Values are presented as number (% within each outcome group). Each patient with a single UPC contributed one identified microorganism.

Abbreviations: Coag(−) *staphylococcus* = coagulase‐negative *Staphylococcus species*, *E. coli* = *Escherichia coli* , *K. pneumoniae* = *Klebsiella pneumoniae* , *Moraxella* sp. = *Moraxella* species, MRSA = methicillin‐resistant *Staphylococcus aureus* , *P. aeruginosa* = *Pseudomonas aeruginosa* , OSSA = oxacillin‐sensitive *Staphylococcus aureus* , *S. aureus* = *Staphylococcus aureus* , *S. epidermidis* = *Staphylococcus epidermidis* , *S. mitis* = *Streptococcus mitis* , UPC = unexpected positive intraoperative culture.

### 3.2 Postoperative Antibiotic Duration and Treatment Failure

Among the 39 patients with a single UPC, the median postoperative antibiotic duration was 14 days (IQR, 5.5–35; range, 3–85 days). Longer postoperative antibiotic duration was associated with treatment failure in patients with a single UPC. Among failures, 85% received antibiotics for > 21 days compared with 31% of successes (*p* = 0.013). Similar associations were observed at the > 14‐day (*p* = 0.003) and > 28‐day (*p* = 0.003) thresholds (Table 4).

**TABLE 4:** Antibiotic duration in a total of 39 patients with a single unexpected positive intraoperative culture (UPC).

Antibiotic duration, *n* (%)	Success (*n* = 32)	Failure (*n* = 7)	*p*
> 7 days	20 (62)	7 (100)	0.077
> 14 days	12 (38)	7 (100)	0.003
> 21 days	10 (31)	6 (85)	0.013
> 28 days	7 (22)	6 (85)	0.003
> 35 days	6 (19)	4 (57)	0.057
> 42 days	4 (13)	3 (43)	0.094

*Note:* Patients were stratified based on multiple antibiotic duration thresholds (> 7, > 14, > 21, > 28, > 35, and > 42 days), and proportions of treatment success and failure were compared at each cut point.

*p* values < 0.05 were considered statistically significant.

In the univariate logistic regression analysis, antibiotic therapy extending beyond 21 days demonstrated a statistically significant association with treatment failure (OR 28.11, *p* = 0.0188) (Table 5). However, due to the small event counts within exposure categories, this estimate was highly imprecise, as indicated by the wide 95% confidence interval (2.48–950.3). Therefore, these findings serve strictly as exploratory trends rather than definitive clinical thresholds.

**TABLE 5:** Association between postoperative antibiotic duration and treatment failure in patients with a single unexpected positive intraoperative culture (UPC).

Antibiotic duration	OR	95% CI	*p*
> 7 days	17.13	1.00–294.8	0.0499
> 14 days	__^a^	__^a^	__^a^
> 21 days	28.11	2.48–950.3	0.0188
> 28 days	__^a^	__^a^	__^a^
> 35 days	5.83	0.16–64.14	0.115
> 42 days	5.22	0.58–57.47	0.141

*Note:* Univariate logistic regression of antibiotic duration and treatment failure in 39 patients with 1 UPC. Odds ratios (ORs), 95% confidence intervals (CIs), and *p* values are presented for multiple antibiotic duration thresholds.

^a^ Model estimates could not be obtained because of sparse event counts and complete separation.

### 3.3 Failure‐Free Survival

The median follow‐up duration was 51 months (IQR, 26–60; range, 1–149 months). Treatment failure occurred in 7 of 39 patients (17.9%) in the 1 UPC group and 5 of 12 patients (41.7%) in the ≥ 2 UPC group. Overall, 12 treatment failures occurred, while 39 patients were censored; 22 were administratively censored at 60 months and 17 were censored at their last available follow‐up before 60 months. Seven patients were lost to follow‐up before 1 year and were excluded before formation of the final study cohort; no additional patients were lost to follow‐up among the 51 included patients. Failure‐free survival differed significantly between the groups on Kaplan–Meier analysis (log‐rank *p* = 0.024) (Figure 2). At approximately 60 months, estimated failure‐free survival was 82.1% in the 1 UPC group and 58.3% in the ≥ 2 UPC group.

**FIGURE 2:**
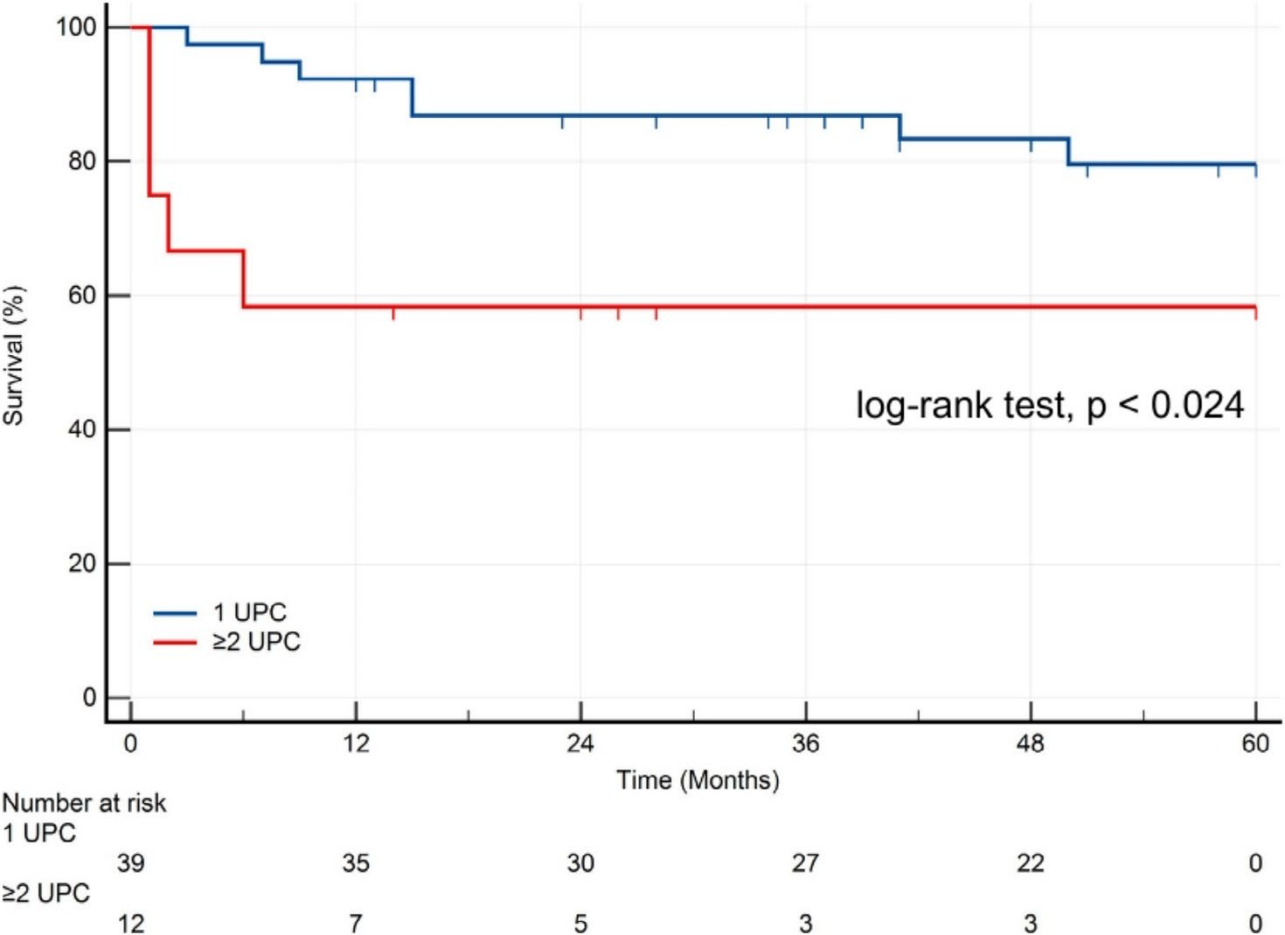
Kaplan–Meier survival analysis comparing failure‐free survival between patients with a single unexpected positive intraoperative culture (1 UPC) and those with multiple UPCs (≥ 2 UPCs). Patients with a single UPC showed significantly higher survival (log‐rank test, *p* = 0.024).

## 4 Discussion

### 4.1 Postoperative Antibiotic Duration and Treatment Failure

In this study, we evaluated the association between postoperative antibiotic duration and treatment failure in patients with UPCs following aseptic revision THA and TKA, with a specific focus on single positive cultures (1 UPC). Prolonged antibiotic use was associated with higher failure rates, suggesting that extended therapy may not provide universal benefit. This observation aligns with previous work demonstrating that even a single UPC may not be clinically benign. Milandt et al. [9] reported increased all‐cause and infection‐related re‐revision in patients with 1 UPC, while Purudappa et al. [11] and Caternicchia et al. [10] similarly highlighted the uncertainty surrounding low‐virulence organisms such as *S. epidermidis* , where failure occurred despite prolonged antibiotic therapy.

The microorganism distribution did not show a clear concentration of high‐virulence organisms in the failure group. Given the small number of failures, the influence of organism virulence on outcome could not be reliably assessed. Kayani et al. [12] reported excellent outcomes using a standardized protocol requiring ≥ 3 concordant cultures and ≥ 3 months of targeted antibiotics; however, those results primarily apply to high‐certainty infections and do not address the clinical ambiguity associated with isolated UPCs. Our study focuses on this diagnostic gray zone, where the role and duration of antibiotics remain unclear.

Regarding postoperative antibiotic management, Simon et al. [13] reported that patients with UPCs who received postoperative antibiotics had no lower septic or aseptic re‐revision rates than matched culture‐negative revision controls, despite a median antibiotic duration of 41 days. However, their study did not directly compare different antibiotic durations. These findings suggest that the benefit of postoperative antibiotic treatment in UPC cases remains uncertain.

Although single UPCs have traditionally been regarded as contamination, accumulating evidence challenges this assumption. Both Milandt et al. [9] and Saleh et al. [3] observed increased recurrence or re‐revision in patients with 1 UPC, especially when virulent organisms were present. Conversely, Neufeld et al. reported that no patient with a single UPC who did not receive antibiotics developed subsequent PJI caused by the same microorganism, highlighting the uncertainty surrounding the clinical significance of an isolated positive culture [14]. These data emphasize that culture results must be interpreted within a broader clinical and microbiologic context rather than relying solely on numeric thresholds. Similar uncertainty has been reported in other orthopedic settings. Systematic reviews of presumed aseptic revision spine surgery and long‐bone nonunion have found unexpected positive cultures to be relatively common, with *Cutibacterium acnes* and coagulase‐negative staphylococci among the predominant organisms, while the optimal management remains unclear [15, 16, 17].

Our results raise questions regarding the routine use of prolonged postoperative antibiotics in patients with a single UPC. While courses longer than 6 weeks are often prescribed, shorter durations may be appropriate for patients with favorable host factors or low‐virulence organisms. Conversely, patients requiring antibiotics beyond 21 days may warrant closer monitoring or earlier surgical intervention, as the need for extended therapy likely serves as a clinical marker of greater underlying infection severity and higher failure risk. Although longer thresholds (> 28, > 35, and > 42 days) showed similar trends, significance was limited by sample size.

### 4.2 Interpretation of Synovial and Clinical Findings

Concerns regarding overtreatment parallel findings from diagnostically ambiguous or culture‐negative infections. Xu et al. [18] reported similar treatment success between culture‐negative and culture‐positive PJI, but a higher rate of antibiotic‐related complications in the culture‐negative group, in which broad‐spectrum empirical regimens were commonly used. Deirmengian et al. reported that synovial fluid cultures may yield false‐positive results, emphasizing the need to interpret microbiological findings together with other clinical and laboratory findings [19]. Our findings regarding synovial fluid markers did not differentiate between success and failure. However, these results must be interpreted with extreme caution, as they are based on a very small, nonrandom subset of patients, which limits our ability to draw definitive conclusions about the predictive value of these biomarkers.

These findings underscore the need for careful antibiotic stewardship in microbiologically uncertain scenarios. Importantly, the observed association between prolonged antibiotics and failure may partly reflect confounding by indication. Clinicians may have extended therapy in patients perceived to be at higher infection risk—such as those with elevated inflammatory markers, intraoperative purulence, or high‐virulence organisms—making longer antibiotic courses a surrogate marker of disease severity rather than a causal risk factor. Thus, prolonged therapy should be interpreted cautiously in light of potential selection bias and unmeasured confounding. Available proxy measures, including preoperative inflammatory markers and revision indication, were examined in univariate analyses. However, detailed intraoperative findings and surgeon‐specific rationale for antibiotic treatment were not consistently available in a standardized form, and multivariable adjustment was not performed because of the limited number of failure events.

### 4.3 Failure‐Free Survival According to Culture Number

Finally, failure‐free survival was significantly higher in patients with a single UPC compared to those with ≥ 2 UPCs, reaffirming the prognostic relevance of culture number. Due to the limited sample size of the ≥ 2 UPC cohort (*n* = 12), further stratification by antibiotic duration was not statistically feasible for this subgroup. Nonetheless, the clinical significance of a single UPC remains uncertain. While Saleh et al. [3] reported an increased risk of subsequent infection in selected patients with a single positive culture, Vargas‐Reverón et al. [20] found no significant increase in five‐year re‐revision risk associated with UPCs.

### 4.4 Strengths and Limitations

This study focused on a clinically ambiguous subgroup of presumed aseptic revision arthroplasties with a single UPC and used a strict definition of treatment failure based on infection‐related re‐revision. Patient‐level antibiotic and microbiological data were also available, and failure‐free survival was assessed for up to 60 months. However, several limitations should be acknowledged. It is a single‐center retrospective analysis, which may limit generalizability. The small sample size—particularly in the failure group—reduces statistical power. A formal a priori power calculation was not performed because of the retrospective study design and fixed cohort size. With only seven failures, multivariable modeling was omitted to avoid severe overfitting and quasi‐complete separation. Furthermore, screening multiple antibiotic cut‐points increases Type I error rates, rendering these threshold findings strictly exploratory. Because the objective was to evaluate antibiotic‐duration patterns and outcomes among patients with UPCs, culture‐negative revisions were excluded and were not used as a control group. Therefore, the findings should be interpreted within a UPC‐positive cohort rather than as a comparison with culture‐negative revisions. Postoperative antibiotic duration was not standardized and may have been influenced by clinician judgment, introducing confounding by indication and making it unclear whether prolonged therapy contributed to failure or simply reflected higher perceived risk. To avoid potential circularity, the primary outcome was defined strictly as infection‐related re‐revision after the index procedure. However, incorporation bias cannot be excluded because the decision to perform re‐revision may have been influenced by knowledge of the positive culture results and subsequent antibiotic treatment. Furthermore, synovial fluid analysis was available in only 13 of the 39 patients in the single‐UPC subgroup because arthrocentesis was performed selectively based on clinical suspicion. This small, nonrandom sample limits the statistical power to detect meaningful differences in synovial markers, and these findings should therefore be considered exploratory. Finally, we did not stratify antibiotic selection, dosing intensity, or host immunologic variability, all of which may have affected microbiological findings and their interpretation.

The retrospective design also introduces potential selection bias and variability in antibiotic documentation, sampling, and postoperative management. Residual and unmeasured confounding may still have influenced the results.

## 5 Conclusion

In conclusion, prolonged postoperative antibiotic duration was associated with higher failure rates in patients with a single unexpected positive intraoperative culture. However, this association may reflect underlying infection severity rather than a causal effect of antibiotic duration. Future prospective or stratified studies are needed to determine which patients benefit from extended treatment and to guide more individualized, risk‐based management.

## Author Contributions

**Hao‐Chang Hsieh:** writing – original draft, data curation. **Feng‐Chih Kuo:** conceptualization, supervision, writing – original draft, investigation, project administration. **Allen Herng Shouh Hsu:** data curation, formal analysis, resources. **Shih‐Hsiang Yen:** data curation, resources. **Po‐Chun Lin:** data curation, resources. **Cheng‐Ta Wu:** writing – review and editing, conceptualization, investigation, funding acquisition, project administration.

## Funding

This work was partially supported by Kaohsiung Chang Gung Memorial Hospital, Taiwan, China (Grant Number: CMRPG8N1281 to C.‐T. Wu).

## Ethics Statement

This study adhered to the Declaration of Helsinki and this study was approved by the ethics committee of the Chang Gung Medical Foundation, with the approval reference number (IRB No.: 202101507B0C601).

## Consent

Informed consent was waived by the ethics committee of Chang Gung Medical Foundation.

## Conflicts of Interest

The authors declare no conflicts of interest.

## Data Availability

The data that support the findings of this study are available from the corresponding author upon reasonable request.
